# Diagnosis of antiphospholipid syndrome in routine clinical practice

**DOI:** 10.1177/0961203312460722

**Published:** 2013-01

**Authors:** C Gardiner, J Hills, SJ Machin, H Cohen

**Affiliations:** 1 Department of Haematology, University College London Hospitals NHS Foundation Trust, London; 2 University College London, London; 3 Nuffield Department of Obstetrics and Gynaecology, University of Oxford, Oxford, UK

## Abstract

The updated international consensus criteria for definite antiphospholipid syndrome (APS) are useful for scientific clinical studies. However, there remains a need for diagnostic criteria for routine clinical use. We audited the results of routine antiphospholipid antibodies (aPLs) in a cohort of 193 consecutive patients with aPL positivity-based testing for lupus anticoagulant (LA), IgG and IgM anticardiolipin (aCL) and anti-ß_2_glycoprotein-1 antibodies (aß_2_GPI). Medium/high-titre aCL/aβ_2_GPI was defined as >99th percentile. Low-titre aCL/aβ_2_GPI positivity (>95^th ^< 99^th^ percentile) was considered positive for obstetric but not for thrombotic APS. One hundred of the 145 patients fulfilled both clinical and laboratory criteria for definite APS. Twenty-six women with purely obstetric APS had persistent low-titre aCL and/or aβ_2_GPI. With the inclusion of these patients, 126 of the 145 patients were considered to have APS. Sixty-seven out of 126 patients were LA-negative, of whom 12 had aCL only, 37 had aβ_2_GPI only and 18 positive were for both. The omission of aCL or aβ_2_GPI testing from investigation of APS would have led to a failure to diagnose APS in 9.5% and 29.4% of patients, respectively. Our data suggest that LA, aCL and aβ_2_GPI testing are all required for the accurate diagnosis of APS and that low-titre antibodies should be included in the diagnosis of obstetric APS.

## Introduction

The antiphospholipid syndrome (APS) is characterized by thrombotic and/or pregnancy morbidity associated with the presence of persistent antiphospholipid antibodies (aPLs).^1^ There are many other clinical manifestations associated with persistent aPL (including immune thrombocytopenia, livedo reticularis, migraine, valvular heart disease and cognitive dysfunction), and, while these conditions are not considered diagnostic for APS, they are frequently encountered and require clinical attention.

The updated international consensus (Sydney) classification (ICS) criteria for definite antiphospholipid syndrome^1^ require the presence of a lupus anticoagulant (LA) and/or IgG or IgM anticardiolipin antibodies (aCL) present in medium or high titre (i.e. >40 GPL or MPL or >99^th^ percentile), and/or anti-β_2_glycoprotein-1 (aβ_2_GPI) (IgG and/or IgM) >99^th^ percentile. These aPL should be persistent, defined as being present on two or more consecutive occasions at least 12 weeks apart. The international consensus criteria were originally designed for scientific clinical studies and were never intended for diagnostic use. Consequently, there remains a need for firm diagnostic criteria for routine clinical use, which may differ from these.

The criteria for the laboratory diagnosis of APS remain controversial. It has been proposed by some that the Sydney laboratory criteria should be modified such that testing for aβ_2_GPI should be limited to measurements of IgG aβ_2_GPI only and testing for aCL should be omitted.^2^ The basis for this is that in a systematic review, LA showed the highest strength of association with thrombotic complications^3,4^ and IgG but not IgM aβ_2_GPI was associated with thrombosis. In addition, Opatrny et al. reported in a meta-analysis that LA was also most strongly associated with late (>13 and <24 weeks) recurrent fetal loss.^4^ Galli et al.^3^ also drew attention to the need to produce guidelines, which were subsequently published,^5^ attempting to standardize more clearly the criteria for the detection of LA.

Others have argued that it is premature to consider reducing the number of assays used in the diagnosis of APS. The systematic review by Galli et al.^3^ referred to above also suggested that medium- or high-titre IgG aCL may represent a possible risk factor for thrombosis. We and others have previously reported that omission of aCL testing from the clinical investigation of APS could lead to a failure to diagnose the syndrome in a proportion of patients,^6–8^ and, in a multicentre prospective European women cohort, isolated aCL and/or aβ_2_GPI positivity was found in a proportion of women with obstetric APS.^7^ The cut-off for serological positivity is also contentious. It has been reported that women with obstetric APS (without systemic thromboembolism) have lower aCL antibody titres than patients with a thrombotic history.^9^ Data from a retrospective cohort study^10^ and also in the prospective European cohort^7^ suggest that low-titre aCL, defined as those between the 95^th^ and 99^th^ percentiles rather than the 99^th^ percentile as suggested in the ICS criteria, are of clinical significance for women with purely obstetric APS.

Wahl et al. suggested that modifications of the serological criteria for the diagnosis of APS should in the future be based on new data and on appropriate systematic reviews.^8^ The proposed entity of seronegative APS, where patients have characteristic clinical manifestations of APS but lack conventional serological markers, has also been given consideration in classification criteria for APS.^11^ We report on serological criteria in a cohort of patients diagnosed to have APS, based on a comprehensive methodological approach which included testing for LA as well as IgG and IgM aCL and aβ_2_GPI.

### Methods

#### Patients and samples

We audited data on routine aPL testing retrospectively from a cohort of 193 consecutive patients attending the thrombosis and haemostasis, recurrent miscarriage or high-risk antenatal clinics at UCLH, who had persistent aPL positivity based on testing for LA, IgG and IgM aCL and aβ_2_GPI on two or more consecutive occasions at least 12 weeks apart. Case ascertainment was based on review of the clinic letters of all patients attending the clinics specified above. These clinic letters were saved prospectively in a dedicated area on the hospital electronic records system so that they were all immediately retrievable.

In patients with thrombotic APS, *high-risk* patients have been recognized in the literature to include those who experience recurrent venous events or arterial thrombosis.^12,13^ However, there are no agreed published definitions for the categorization of the clinical severity of thrombotic, or obstetric, APS. In this audit, *high-risk APS* was defined as the presence of any of the following clinical scenarios: recurrent objectively diagnosed thrombotic events; thromboses in both venous and arterial sites; both early and late pregnancy morbidity as defined in the Sydney clinical criteria for APS^1^; both pregnancy morbidity and thrombotic events; and/or thrombosis or pregnancy morbidity whilst receiving anticoagulant therapy. The remainder were categorized as *lower-risk*.

Venous blood for LA was collected into 5 ml tubes containing one-tenth volume 0.105 M tri-sodium citrate (e.g. Vacutainer®, Becton Dickinson, Plymouth, UK) using 19 or 21 gauge needles and minimal stasis. For LA, platelet-poor plasma was prepared by double centrifugation at room temperature at 2000 *g* for 15 minutes, and frozen in aliquots at −80°C until assayed. aβ_2_GPI antibodies and aCL were performed on serum.

#### Antiphospholipid antibody assays

All patients in the study cohort had persistent aPL–that is, aPL were present on two or more consecutive occasions at least 12 weeks apart.

aCL assays were performed using an in-house assay employing 10% fetal calf serum as a blocking agent^14^ based on the work of Loizou et al.^15^ and standardized using the polyclonal ‘Harris’ standards.^15,16^ The Sydney laboratory criteria state that medium- or high-titre aCL are those above the 99^th^ percentile or 40 GPLU/MPLU. In this study, aCL positivity was defined as: medium/high titre (99^th^ percentile) >20 GPLU/MPLU; low-titre (95–99^th^ percentile) IgG > 5 GPLU/MPLU. For patients with thrombotic APS, the 99^th^ percentile cut-off was used to define aPL positivity. However, a number of studies have suggested the 99^th^ percentile is too insensitive for clinical use for women with fulfilling Sydney clinical criteria for obstetric APS (and no systemic thrombotic manifestations)^7,9,10,17^ so the 95^th^ percentile value was used to define in these patients.

β_2_GPI were measured using a commercial kit (Axis; Shield Diagnostics, Dundee, UK), based on a method developed in our department.^18^ aβ_2_GPI positivity was defined as: low-titre (95–99^th^ percentile) IgG > 3.5 units, IgM > 3.0 units; medium/hightitre (99^th^ percentile) >15 units.

##### Reference ranges for aCL and aβ_2_GPI were obtained from 240 normal healthy volunteers

LA testing and derivation of the local reference range were performed according to ISTH^19^ and BCSH guidelines^20^ current at that time, prior to publication of the updated ISTH criteria.^5^ Briefly, samples were screened using the activated partial thromboplastin time (Pathromtin SL; Dade Behring, Marburg, Germany) and appropriate mixing studies. LA activity was confirmed using a dilute Russell's viper venom time (DRVVT) employing a platelet neutralization procedure (Pathway Diagnostics, Dorking, UK). Patients receiving warfarin were tested using the Taipan venom time as previously described.^20,21^ All patients had at least one DRVVT performed whilst not receiving warfarin.

#### Statistical analysis

Non-parametric statistical methods were used throughout. The Mann–Whitney *U* test was used to test for statistically significant differences between medians. Linear-by-linear association was assessed using the Mantel–Haenszel chi-squared test. Statistical significance was defined as *p* < 0.05. Statistical analyses were performed using SPSS 16.0 (SPSS Inc. Chicago, IL).

## Results

**Figure 1:**
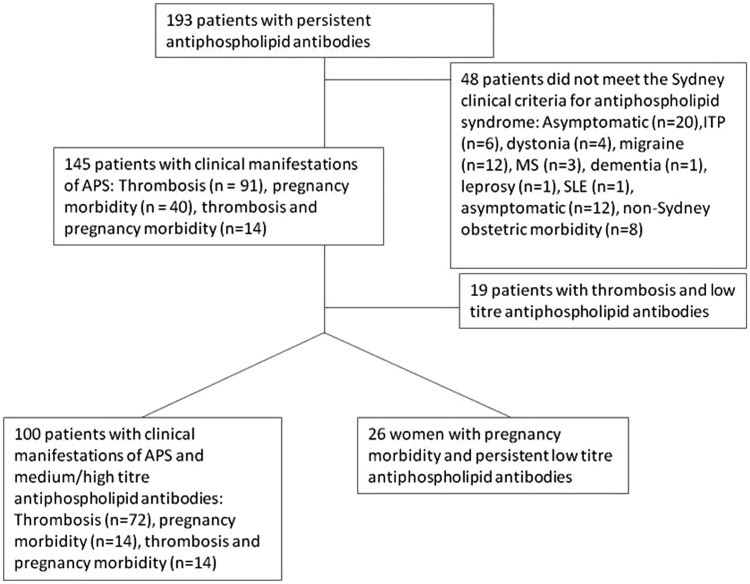
Flowchart showing patient selection according to Sydney clinical criteria for APS.

Figure 1 shows a flowchart of patient selection according to Sydney clinical criteria for APS, with clinical diagnoses summarized here. One hundred and forty-five of the cohort of 193 patients had the following clinical manifestations which fulfilled the Sydney clinical criteria 1: thrombosis (total 91; arterial 33; venous 48; both arterial and venous 10); early and/or late pregnancy morbidity as defined in the Sydney clinical criteria for APS (54), with 14 of these 54 exhibiting both thrombotic and obstetric manifestations. Nineteen of the 145 had underlying autoimmune disease (including 11 with systemic lupus erythematosus (SLE), two of whom also had ITP, and a further two with ITP). The remaining 48 of the 193 patients, who did not meet the Sydney clinical criteria, had the following diagnoses: immune thrombocytopenia (6), dystonia (4), migraine (12), multiple sclerosis (3), dementia (1), leprosy (1) or SLE (1). Eight were referred with a history of obstetric morbidity which did not meet the Sydney criteria and 12 were asymptomatic. These 48 patients will not be discussed further.

**Figure 2:**
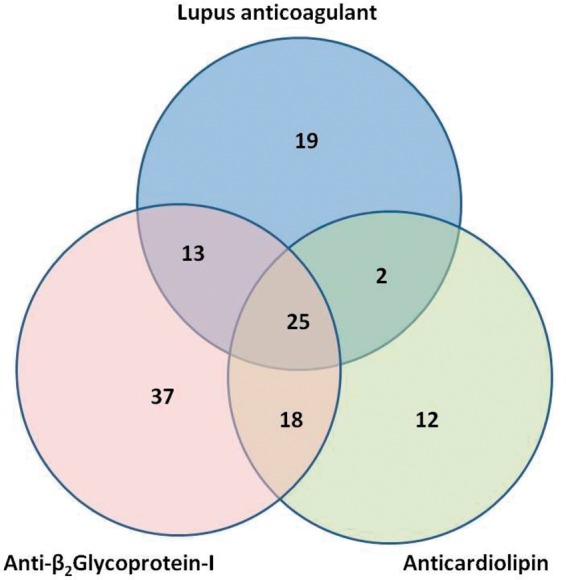
Distribution of antiphospholipid positivity in 126 patients with antiphospholipid syndrome as detailed in Figure 1.

The distribution of aPL positivity is summarized in Figure 2 and by clinical diagnoses in Table 1. Of the 145 patients who fulfilled the Sydney clinical criteria for APS, 100 also fulfilled the Sydney laboratory criteria; that is, if aCL and/or aβ_2_GPI were present their levels were greater than the 99^th^ percentile. A further 26 women who fulfilled clinical criteria for purely obstetric APS (and without a history of thrombosis) had persistent low-titre aCL and/or aβ_2_GPI; that is, above the 95^th^ percentile but below the 99^th^ percentile, in the absence of LA. Thus a total of 126/145 patients were considered to have APS according to our local criteria. Sixty-seven of these 126 patients were LA-negative, of whom 12 had aCL positivity only, 37 had aβ_2_GPI positivity only, and 18 were positive for both aCL and aβ_2_GPI. Consequently, omission of aCL or aβ_2_GPI from the laboratory investigation of APS would have resulted in the failure to diagnose APS in 9.5% and 29.4% of patients, respectively. Twenty-two of these 67 patients had *high-risk APS* as defined in the Methods section above, of whom 11.5% had single positivity for aβ_2_GPI or aCL. IgM aCL and/or aβ_2_GPI antibodies (1 aCL, 25 aβ_2_GPI, 3 aCL and aβ_2_GPI), alone or in association with IgG antibodies, were found in 53.7% (29/54) of women who fulfilled Sydney clinical criteria for purely obstetric APS.

**Table 1:** The distribution of antiphospholipid positivity by clinical diagnoses

	Pregnancy morbidity (*n* = 40)		Thrombosis (*n* = 105^a^)	
	>99^th^ percentile	>95^th ^< 99^th^ percentile	>99^th^ percentile	>95^th^ < 99^th^ percentile
**IgG aβ_2_GPI**	4 (10.0%)	6 (15.0%)	30 (28.6%)	40 (38.1%)
**IgM aβ_2_GPI**	6 (15.0%)	23 (57.5%)	25 (23.8%)	59 (56.2%)
**IgG aCL**	4 (10.0%)	13 (32.5%)	23 (21.9%)	45 (42.9%)
**IgM aCL**	0 (0.0%)	4 (10.0%)	10 (9.5%)	21 (20.0%)
**LA positivity**	6 (15.0%)^b^		53 (50.5)^b^	

a Includes 14 women with both thrombosis and pregnancy morbidity.

The number (percentage) of positive tests and median antibody levels (>99^th^ or > 95^th ^< 99^th^ percentile) are given or each clinical group.

b Chi-squared: patients with pregnancy morbidity versus those with thrombosis *p* = 0.0002.

aβ_2_GPI: anti-beta_2_ glycoprotein-1 antibodies; aCL: anticardiolipin antibodies; LA: lupus anticoagulant.

**Figure 3:**
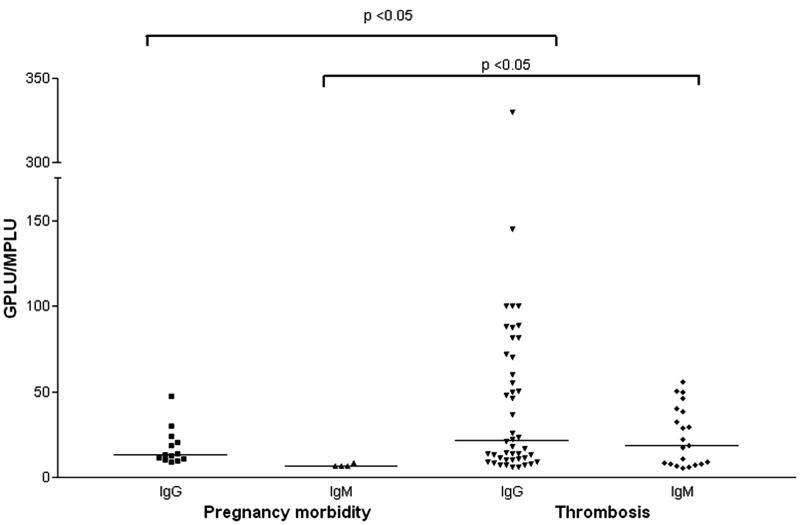
Anticardiolipin (aCL) results by clinical history. Pregnancy morbidity (*n* = 40), Thrombosis (*n* = 105, including 14 with both thrombosis and pregnancy morbidity). All aCL values above the 95^th^ percentile are shown. Horizontal bars represent the median titres.

**Figure 4:**
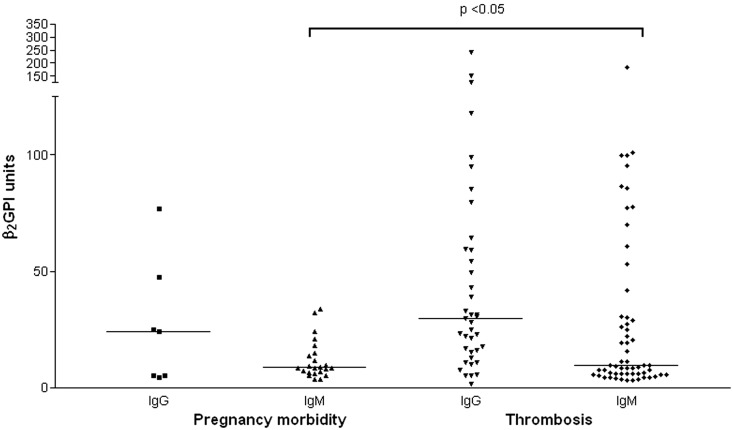
anti-β_2_glycoprotein-1 (aβ_2_GPI) results by clinical history. Pregnancy morbidity (*n* = 40), thrombosis (*n* = 105, including 14 with both thrombosis and pregnancy morbidity). All aβ_2_GPI values above the 95^th^ percentile are shown. Horizontal bars represent the median titres.

IgG aCL and IgM aCL levels (Figure 3) and IgM aβ_2_GPI levels (Figure 4) were significantly higher in patients with a history of thrombosis than in women with a history of purely obstetric APS (*p* < 0.05). Similarly the rate of LA positivity was also significantly higher in patients with a history of thrombosis compared with those with obstetric APS alone (50.5% v 15%; *p* = 0.0002).

*High-risk APS,* as defined in the Methods section, was observed in 60 of the 145 (41%) patients fulfilling the Sydney clinical criteria for thrombosis or obstetric APS. The number of positive tests (as defined by the Sydney laboratory criteria) was significantly associated with clinical severity in patients who fulfilled both clinical and laboratory Sydney criteria: 42.2% with one positive test, 41.2% with two positive tests; and 64.7% with three positive tests (*p* = 0.026). In contrast, only 30.0% of patients with low-titre aPL alone had a clinical history consistent with *high-risk APS* as defined in the Methods section*.*

**Figure 5:**
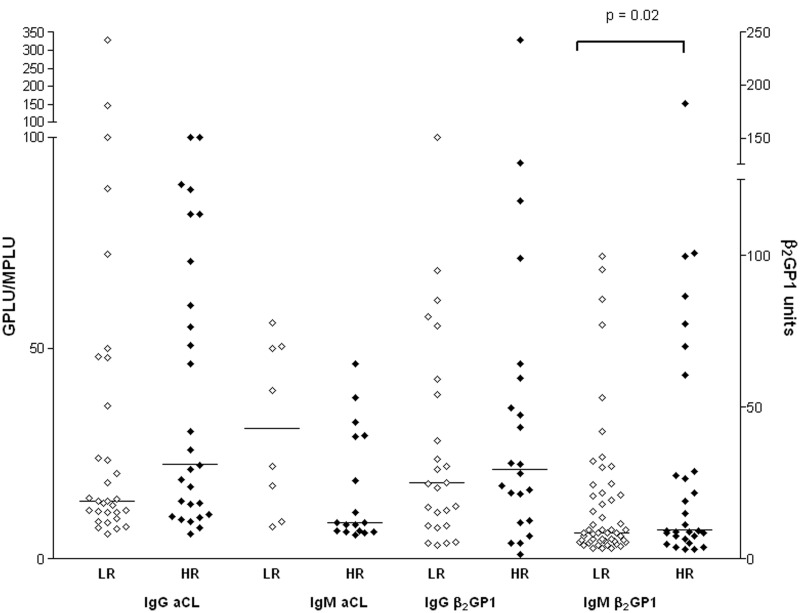
Antiphospholipid antibody results by clinical risk: *low-risk antiphospholipid syndrome (APS) n* = 85, *high-risk APS n* = 60. Horizontal bars represent the median titres.

With regard to the association between positivity for a single aPL test and severity of APS, only the incidence of LA (50.0% v 31.8%, *p* < 0.05) and median titre of IgM aCL (Figure 5) were associated with *high-risk APS* in this cohort. Twenty-five out of 54 (46.3%) women with a history of pregnancy morbidity had clinical histories consistent with *high-risk APS* (as defined in the Methods section) compared to 7/26 (26.9%) of the same group who had low-titre APL.

## Discussion

We report the clinical and laboratory findings of a cohort of 193 patients who had persistently positive tests for aPL. One hundred of these 193 patients had APS as defined by Sydney clinical as well as laboratory criteria^1^; and 126 when women with purely obstetric APS (without a history of thrombosis) associated with low-titre aCL and/or aβ_2_GPI were also included as suggested in a number of other studies7,9,10,17 although not included in the Sydney laboratory criteria for APS.1.

Our data indicate that omission of testing for aCL or aβ_2_GPI from the clinical investigation of APS would have led to a failure to diagnose the syndrome in 9.5% and 29.4% of patients respectively. Although the term *high-risk* in relation to patients with thrombotic APS now appears in the literature,^12,13,22^ there are no agreed published definitions for this term in thrombotic or obstetric APS. To assess the clinical relevance of persistent aPL in our cohort, as it is possible that these persistent aPL could have occurred by chance, we categorized patients in our cohort as having *high-risk* or *lower-risk* APS as detailed in the Methods section and correlated aPL results with clinical risk. We found, in agreement with previous reports, that LA as well as triple positivity (that is, aCL,^23^ aβ_2_GPI and LA) were associated with *high-risk APS*.^3,22,24,25^ Furthermore, 11.5% of patients with single positivity for aβ_2_GPI or aCL (including IgM aCL) had *high-risk APS*, highlighting the importance of inclusion of these tests in the diagnosis of APS, as previously reported.^7,26−28^

Over 50% of women with clinical features of obstetric APS, but no thrombosis, had low-titre aCL and/or aβ_2_GPI in the absence of LA. Approximately 27% of these patients had clinical features of *high-risk APS*. The association of low-titre aCL or aβ_2_GPI in women with clinical features consistent with Sydney clinical criteria for obstetric APS could have arisen by chance; however, our findings concur with those of Ruffati et al.^9^ who reported that the rate of aCL values between the 99th percentile and 40 GPL units was significantly higher (*p* < 0.0001) in patients with pregnancy morbidity (73.7%) as compared to those with vascular thrombosis (16.9%) and those with both conditions (16.7%), and concluded that the 99th percentile cut-off level appears more sensitive than the >40 GPL value for the diagnosis of APS in women with persistent aCL positivity alone associated with obstetric APS. Low-titre aCL or aβ_2_GPI, in some cases occurring as an isolated phenomenon, also appeared to be clinically significant in an analysis of laboratory findings in a prospective European cohort of women with obstetric APS.^7^ Furthermore, in clinical studies, persistent low-titre aCL were associated with a >90% fetal loss rate in untreated pregnancies of women with recurrent miscarriage and aPL, and with significantly improved pregnancy outcome following treatment with low-dose aspirin or heparin and aspirin.^10,29–31^ Our data also support the inclusion of low-titre IgG and IgM aCL and aβ_2_GPI antibodies in the diagnosis of APS in women with a history of clinical obstetric APS, where accurate diagnosis has therapeutic implications during pregnancy with potentially far-reaching adverse clinical consequences in an infant who may suffer long-term physical disability and mental impairment as a result of placental-mediated obstetric morbidity such as intrauterine growth restriction, early onset pre-eclampsia or placental insufficiency/abruption.

The pattern of aPL positivity in our cohort differs from that in some published cohort studies. There may be several reasons for this. First, the aCL ELISA used in this study does not contain additional purified β_2_GPI and uses fetal calf serum as a blocking agent. Despite efforts to standardize solid phase assays for aCL, agreement between laboratories remains poor.^32,33^ The source of cardiolipin and technique used to coat the microtitre plates is known to affect results.^33^ Some methods employ fetal calf serum as a blocking agent, while others use cardiolipin saturated with human β_2_glycoprotein-1 as the solid phase antigen. The situation for aβ_2_GPI antibody tests is marginally better, with some agreement between most assays.^34,35^ However, different β_2_GPI purification methods are known to give rise to inter-assay variation.^35,36^ It should be noted that when human β_2_GPI is added to aCL assays there is understandably a high degree of agreement between aCL and aβ_2_GPI assays^37^ and this may have led to the suggestion that aβ_2_GPI are not present in patients who are negative for aCL. Conversely, some have argued that aCL assays detect clinically significant antibodies which are not detected by aβ_2_GPI assays.^4,6,7^ Secondly, a significant proportion of our cohort (37%) had purely obstetric APS whereas many published studies have included only patients with a history of thrombosis. Much of the published literature on aPL phenotypes in APS relates to patients with SLE-associated APS, whereas in the patients in our cohort only a minority (19/145) had SLE, as the study cohort comprised patients who had predominantly presented to haematology rather than rheumatology clinics.

In selecting the 95^th^ percentile as a cut-off value for aCL and aβ_2_GPI in purely obstetric APS, a small number of false positives are likely to result. However, if the 99^th^ percentile were used as a cut-off in these women, many cases of obstetric APS are likely to be missed. The role of IgM aβ_2_GPI testing in the diagnosis of APS remains unclear and it considered to have little clinical utility for thrombotic APS.^38^ In our cohort >50% of women with obstetric APS had low-titre IgM aCL and/or aβ_2_GPI (alone or in association with IgG antibodies). Given the favourable risk/benefit ratio of heparin/aspirin treatment during pregnancy for obstetric APS it appears reasonable, at present, to offer this treatment during pregnancy to women with low-titre aPL associated with obstetric APS. However, future studies into the clinical significance and management of low-titre aPL in obstetric APS would be useful.

In conclusion, these data suggest that LA, IgG and IgM aCL and aβ2GPI testing are all required for the accurate diagnosis and assessment of prognosis of APS in routine clinical practice. Furthermore, as laboratory tests for aPL remain poorly standardized internationally, it is prudent to retain testing for LA as well as aCL and aβ_2_GPI for APS diagnosis. Our data also suggest that low-titre aCL and aβ_2_GPI should be included in the laboratory criteria for diagnosis of purely obstetric APS. This should be validated in a prospective multicentre study.
